# Active Commuting to University and its Association with Sociodemographic Factors and Physical Activity Levels in Chilean Students

**DOI:** 10.3390/medicina55050152

**Published:** 2019-05-17

**Authors:** Yaira Barranco-Ruiz, Carolina Cruz León, Emilio Villa-González, Ximena Palma Leal, Palma Chillón, Fernando Rodríguez-Rodríguez

**Affiliations:** 1 PROmoting FITness and Health through Physical Activity (PROFITH) Research Group, Department of Physical Education and Sports, Faculty of Sport Sciences, University of Granada, Camino de Alfacar, 21, 18071 Granada, Spain; evilla@ugr.es (E.V.-G.); pchillon@ugr.es (P.C.); 2 IRyS Research Group, School of Physical Education, Pontificia Universidad Católica de Valparaíso, Av. El Bosque 1290, Viña del Mar 2530388, Chile; carolina.cruz.irys@gmail.com (C.C.L.); fernando.rodriguez@pucv.cl (F.R.-R.); 3 Departamento de Educación Física, Deportes y Recreación DEFIDER, Universidad Técnica Federico Santa María, Valparaíso 2390123, Chile; ximena.palmaleal@gmail.com

**Keywords:** young adulthood, active transport, physical activity, health promotion, university students

## Abstract

*Background and Objectives:* Active commuting to and from university (ACU) could be a strategy to increase physical activity levels (PA) and promote health in young university students. We aimed to a) examine the patterns of commuting to university in Chilean students; b) the association between the mode of commuting to and from university and socio-demographic factors and PA-levels. *Materials and Methods:* A total of 496 university students (21.6 ± 2.4 years old) from two universities from Valparaíso (central coast of Chile) participated in this study. Personal data, home address, socio-economic status, PA, and the usual mode of commuting to and from the university were self-reported by a questionnaire. The commute distances were objectively measured using Google-Maps-software. Associations were examined using binary logistic regressions. *Results:* The main mode of commuting was by bus (to university: 55.2% vs. from university: 59.3%; *p* < 0.001). The least used mode was cycling (1.4% to and from university). Students living >5-km from university were less active commuters than those living in closer distances: (2–5 km, odds ratio (OR): 4.424, 95% and 95% confidence intervals (CI): 2.443–8.011, *p* < 0.001; 2 km, OR: 143.052, 95% CI: 55.154–371.030, *p* < 0.001). Students with low PA-levels were less active commuters than those with medium (OR: 1.446; 95% CI: 0.864–2.421; *p* = 0.160) or higher levels (OR: 1.880; 95% CI: 1.880–1.094; *p* = 0.022). Students who lived between 2 and 5 km, presented a significant association to be active commuters when they showed medium PA-levels (OR: 5.244, 95% CI: 1.358–20.246; *p* = 0.016). *Conclusions:* Chilean university students from Valparaíso are mainly passive commuters using public transport as the main mode of commuting to and from university; longer distances from home to the university are associated with low PA levels. ACU in distances between 2–5 km (mainly walking) could contribute to having medium PA-levels in Chilean university students. Thus, promoting the ACU walking to and from the university in such distances could be an effective strategy to increase the overall PA levels in Chilean university students.

## 1. Introduction

Insufficient physical activity is a crucial risk factor for noncommunicable diseases (NCDs) and is directly associated with overweight and obesity. For that reason, it is considered one of the top 10 risk factors for death worldwide. Insufficiently active people have a 20% to 30% increased risk of death compared to sufficiently active individuals. Correspondingly, more than 80% of the world’s adolescent population are insufficiently physically active [1]. The American countries are most seriously harmed by the overweight epidemic are USA, Mexico, and Chile [2]. In Chile, more than 40% of adolescents have a BMI (body mass index) corresponding to overweight or obesity, and the average physical activity (PA) per week is 4.3 h, which classifies this group as sedentary according to the WHO (World Health Organization) [3]. This decline in physical activity is partially due to inactivity and sedentary behavior during the daily routine (job tasks, home activities, etc.). Likewise, an increase in the use of “passive” modes of transportation also contributes to insufficient physical activity [1].

Active commuting (AC) is a daily behavior considered as an opportunity to create a healthy habit, increasing PA-levels, and reducing different cardiovascular risk factors in the adult and young population [4,5]. For example, walking 1.9 km in 22 min twice per day, 5 days per week, or cycling at 16 km/h for 11 min, twice per day during 5 days per week while commuting to school or the workplace generates an energy expenditure of 4 MJ. Such habits are recommended to reduce all-cause and cardiovascular mortality in the sedentary population [6]. Nevertheless, it must be considered that these active behaviors have a multifactorial basis because they are associated with personal, family, or environmental factors. Thus, before implementing an intervention based on promoting AC, it is important to analyze the patterns of commuting and its association with the main socio-demographic factors.

One of the more important changeovers in youth life happens during the transition from high school to university [7,8]. University is an ideal context for promoting health-related behaviors and the consolidation of adult behavioral patterns has been related to this setting [9,10,11]. However, university students’ health-related lifestyle is a concern since a decrease in the PA-levels is associated with this period [8,12,13]. University life seems to keep students physically inactive for long periods and this leads to a reduction in the overall PA practice [14] without meeting the recommended PA-levels [15].

There is a wide range of evidence related to commuting to school and the socio-demographic factors and PA-levels associated with children and adolescents [16,17]. However, little documented evidence related to commuting patterns and PA-levels in the university community has been observed [18,19,20,21,22], although, since 2006, the number of Chilean students going to university has doubled, from 668,532 students to 1,161,222 students counted in 2016 by the National Council of Chilean Education (CNED). In addition, Chilean university students spend a long time dedicated to academic tasks, have poor nutrition habits, and insufficient time to engage in PA [23,24]. Therefore, ACU (active commuting to university) could be a strategy to increase the PA-levels in this population, promoting a potential change in their lifestyles in this transition to adulthood. However, under our knowledge, there are no previous studies related to commuting patterns in Chilean university students. Thus, this study aimed to analyze the main modes of commuting to and from university in Chilean students and its relationship with socio-demographic factors and PA-levels.

## 2. Materials and Methods

### 2.1. Participants

A total of 496 university students (21.63 ± 2.43 years old, 68% females) from Valparaíso (central coast of Chile) participated in this study. Students belonged to two different universities: Pontificia Universidad Católica de Valparaíso (PUCV, *n* = 346, 69.8%, Sausalito campus) and Universidad Técnica Federico Santa María (UTFSM, *n* = 150, 30.2%, Central campus). Both campuses were located in Valparaiso hills. The sampling was via convenience. Students from both campuses were voluntarily recruited from special courses of “healthy life” or “recreational program of physical activity”, where there were students from several careers. Those students enrolled between 2010 and 2015, as well as those belonging in the career of physical education or athletes involved in the university leagues were excluded.

### 2.2. Ethics, Consent, and Permission

Each institution approved the study protocol and the written informed consent was obtained from all participants before the study. The Ethical Committee of Pontificia Universidad Católica de Valparaíso approved this study (REF: ccf02052017; approval date: 01-05-2015). All procedures were in accordance with the 1964 Helsinki declaration and its later amendments or comparable ethical standards in studies involving human participants.

### 2.3. Design

This cross-sectional study was conducted from August to November 2015. The participating institutions allowed completing the questionnaire in the classroom. Data were collected during the morning schedule before starting the lessons. Participants were required to complete the questionnaire individually for approximately 20 min. The questionnaire included four sections addressing personal data of the participants, mode of commuting, socioeconomic status data, and PA-levels.

### 2.4. Personal Data

Participants reported their personal data, age, gender, year of admission to the university, and home address as a university student. The years of university life were calculated (i.e., year of the data collection–the year of entrance to university). Distances from home to university were calculated including the postal addresses of both home and university in Google Maps^TM^ and selecting the shortest distance by foot expressed as km, as it has been reported in previous studies [25]. The distances were classified into 3 categories: ≤2 km, 2–5 km, and >5 km [18].

### 2.5. Mode of Commuting

The usual mode of commuting to and from university was assessed by two questions: (a) *How do you usually go from your residence to university?* (b) *How do you usually go from university to your residence?* While response options were walking, by bike, by car, by bus, by motorcycle, other, and, combined (i.e., at least two modes of transport). These questions were similar to those previously used in other studies with similar age-groups [18,20].

For the final analysis, participants were classified as “active commuters” if they commuted to and/or from university in an active mode, such as walking or by bike, and as “passive commuters” if they commuted to and from university in a passive mode, such as by car, bus, or motorcycle. Participants who answered “combined” (go = 1.6%; from = 1.8 %) or “others” (go and from = 5%) were excluded because if they did not indicate the mode of commuting, they could not be classified into neither active commuters nor passive commuters.

### 2.6. Socioeconomic Status

Participants were asked questions from the Mexican Association of Market Research and Public Opinion (AMAI) for socioeconomic levels. The AMAI defines variables about the year of studies of the family’s head and the family housing conditions as: number of lightbulbs at home, number of rooms (without bathrooms), number of baths with shower inside the home, number of cars, type of floor, and type of boiler. Depending on the amount of the possessions, a score is assigned, and then summed and the total points are obtained. A score was assigned to each variable and participants were classified into three categories regarding the socioeconomic status levels: low (0 to 79 points), medium (80 to 174 points), and high (175 to 283 points) [26].

### 2.7. Physical Activity Levels (PA-Levels)

The PA level was assessed including the 7 questions of the short version of the International PA Questionnaire (IPAQ) [27]. The short version of IPAQ asks about 3 types of PA: walking, moderate-intensity activities, and vigorous intensity activities. The final output provides the sum of the duration (in min) and frequency (days) of walking, moderate, vigorous, and total PA [28]. Participants were finally classified according to the Metabolic Equivalent of Task (MET) into high (>1500 MET min/week), medium, (among 600 to 1500 MET min/week, and low PA-levels (<600 MET min/week) [29]. In addition, total MET min/week and total seated min/week were calculated.

### 2.8. Statistical Analysis

Comparisons between the modes of commuting to and from the university were analyzed by the McNemar test. Comparisons between active and passive commuters were analyzed by the chi-square test for qualitative variables and t-student test for quantitative variables. The association between ACU with socio-demographic factors and PA-levels were studied using binary logistic regressions. The dependent variable was the mode of commuting (active vs. passive) and the independent variables were socio-demographic factors (i.e., age, years of university life, distance from home to university, socioeconomic status) and PA-levels. Each independent variable was analyzed in a separate model. All analyses were adjusted for distance (except the analysis of the distance groups), university, and gender. In addition, the odds ratio for ACU associated to PA-levels were analyzed regarding the three different distance groups from home to university (i.e., those living within 2 km, those living between 2 and 5 km, and those living more than 5 km from university); these analyses were adjusted for university and gender. All the analyses were conducted using the SPSS statistical package for MAC (version 22.0; IBM-SPSS, Armonk, NY, USA) and the level of significance was set at *p* < 0.05.

## 3. Results

### 3.1. Mode of Commuting

**Figure 1:**
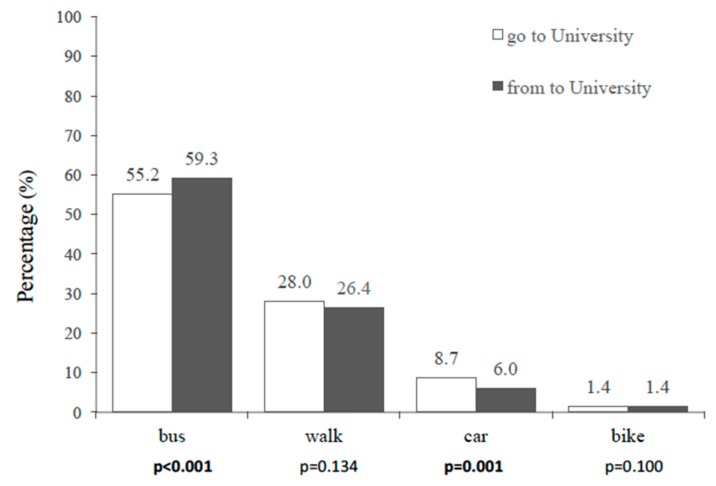
Modes of commuting to and from university in Chilean university students (*p*-value in bold = statistical differences between the modes of commuting to and from university).

The percentage of the usual mode of commuting to and from university is shown in Figure 1. The main mode of commuting was by bus, where the percentage was lower in the commuting to university than from university (55.2% vs. 59.3%; *p* < 0.001). The second most used mode of commuting was walking, without statistical differences between both directions (28.0% vs. 26.4%; *p* = 0.134). The third mode of commuting most used was by car, which presented higher values of commuting to university than from university (8.7% vs. 6.0%; *p* = 0.001). Commuting by bike was the least used mode and presented the same percentages in both directions (1.4%).

### 3.2. Descriptive Data of the Participants

Descriptive data of the socio-demographic factors and PA patterns regarding active and passive commuters are presented in Table 1. There were 67% passive commuters and 33% active commuters. The majority of participants were female (68.0%). The mean age was 21.6 ± 2.4 years, and the mean of university life (years of university experience) was 3.1 ± 1.7 years. The mean (25th, 75th percentiles) distance from home to university was 7.6 (2.8, 17.2) km; for passive commuters, the mean was 10.5 (5.9, 18.2) km and for active commuters, it was 1.1 (0.7, 3.3) km. Most of the sample (47.1%) had a high socioeconomic status; where most of the passive commuters (48.9%) had a high socioeconomic status, whereas most of the active commuters (43.4%) had a medium-high socioeconomic status. Regarding PA patterns, the mean of MET-min/week of the total sample was 2231.4 ± 1898.0; passive commuters showed lower MET min/week (21356.0 ± 1750.4 MET min/week) than active commuters: (2355.4 ± 2198.4 MET min/week). The mean of seated min/week for the whole sample was 2962.2 ± 1787.1. Similar seated min/week were found in both passive (2942.6 ± 1857.3 seated min/week) and active commuters (3009.7 ± 1715.1 seated min/week). Most of the samples (38.3%) showed a medium PA level. Statistical differences between active and passive commuters were found only regarding distance (mean of distance and groups, *p* < 0.001).

### 3.3. Associated Factors with Commuting to University: Socio-Demographic Factors and PA-Levels

Associations between ACU with socio-demographic factors and PA-levels are shown in Table 2. There were significant associations for distance and PA-levels. Students who lived far from university (more than 5 km) were less ACU than those students who lived in closer distances from 2–5 km (OR: 4.424; 95% CI: 2.443–8.011; *p* < 0.001) and less than 2 km (OR: 143.052, 95% CI: 55.154–371.030; *p* < 0.001). Regarding the patterns of PA, students with low PA-levels were less active commuters than those with medium (OR: 1.446; 95% CI: 0.864–2.421; *p* = 0.160) and higher PA-levels (OR: 1.880; 95% CI: 1.880–1.094; *p* = 0.022). 

Additionally, the association between PA-levels and ACU was analyzed separately regarding the distance from home to university (Table 3). There were no significant differences in students who lived within 2 km and further than 5 km from university. There were only significant differences among students living between 2 and 5 km from university; those with medium PA-levels showed a high odds ratio for being active commuters than those with low PA-levels (OR: 5.244, 95% CI: 1.358–20.246; *p* = 0.016).

## 4. Discussion

The present study describes the mode of commuting to and from university in students from the Central Coast of Chile and its association with socio-demographic factors and PA-levels. The bus was the main mode of commuting to and from the university. Accordingly, long distances from home to university, and low PA-levels were positively associated with passive modes of commuting to and from university.

In the current study, 67.0% were passive commuters and 33.0% active commuters to and from university, where the bus was the main mode of commuting followed by walking. Despite the lack of previous studies about ACU in Latin America, studies in other countries such as Spain [18,20] have reported similar results being bus or some public transport the main mode of commuting to and from university. In the Spanish study, the university students reported the train/metro/tram as their first mode of commuting (31.1%), followed by walking (24.3%), car (15.4%), and bus (13.7%). In this case, train/metro/tram were used due to the good connection with the university, since the access time to public transport was low (mean = 6.5 min) [19]. In another study conducted in the University of Western Australia, university students (*n* = 1040) and staff (*n* = 1170) were surveyed reporting two main modes of passive commuting by the students: firstly, the single occupant vehicle (45.85%) and secondly, the public transport (31.90%), such as the bus [20]. In addition, our results reported that the least used active mode of commuting to university was cycling (1.4%). The bike is also the least used mode of commuting in the previous two studies, although the percentages were slightly higher in Spanish (7.5%) and Australian (10.6%) university students. In other cities, such as Hradec Kralove in the Czech Republic, Ghent in Belgium, or Aarhus in Denmark, a high prevalence of any cycling for transport (35.3%, 43.2%, and 62.5%, respectively) compared with other cities (from Cuernavaca, Mexico 1.2% to Olomouc, Czech Republic 18.2%) was shown; however, the data was related to transportation in general but not to transportation to and from the university [30]. Additionally, in another study performed in Belgium, a decrease in active transportation during the transition from high school to higher education was shown [31]; however, active transportation was reported in minutes/week and not as modes of commuting. The lack of studies related ACU in countries with a high rate of bike commuting, as well as non-standardized measures in the studies, makes difficult the comparison with the results of the present study. The low use of bike transportation in the region of Valparaíso could be due to geographical reasons, give the importance of the built environment as a factor associated with bicycling commuting. Most of the population live in very hilly areas with narrow streets that make it difficult to build bike lane and to promote cycling as a commuting mode. Other less hilly cities in Chile report higher use of bicycles, as Rancagua and Los Angeles (4%), Curico (12%), Talca and Chillán (8%), and similar to studies focused on university students. [32].

We did not find associations between the mode of commuting to university and the socio-economic status. One reason might be due to the high percentage of participants classified as middle-high socioeconomic status and the low sensitivity of the socioeconomic measurement. However, there is previous evidence in the scientific literature about this association. In Brazil, AC was associated with low income and living in less economically developed areas in the general population [33]. Accordingly, Spanish urban adolescents’ socio-economic status was inversely related to ACS [34]. ACS in adolescents from California (USA) has also been negatively associated with higher socioeconomic status [35]. Additionally, the use of public transport (bus, metro, train), bike, or walking was also associated with having low incomes [36]. This fact indicates that there may be differences between public and private passive transport and socioeconomic status. Further research concerning this topic among the university population is necessary.

Regarding the distance, students who lived more than 5 km from the university were more likely to be passive commuters than those who lived in shorter distances (<2 km or 2–5 km). According to this, longer distances have been presented in previous literature as the main barrier to being active commuters for children [37], the youth [38,39], adults [4], and university students [20]. In accordance with our results, previous studies in adolescents [40] and university population [18] demonstrated that a distance of under 5 km to school or the workplace were related to AC behaviors. In addition, university students who lived at further distances usually used public transport [20]. A longitudinal study performed in students from 10 to 14 years old [41] established that the threshold distance that students are willing to walk to school increased with age and it was also situated in distances under 5 km (from 1421 m in 10-year students to 3046 m in 14-year students). However, during the transition from adolescence to adulthood the increase in the distance to school/workplace during the study period, which was more than 5 km on average, was associated with decreases in AC [8], especially in males. In our study, the mean distance from home to university was higher in passive commuters than in active commuters (14.9 ± 0.8 km vs. 5.9 ± 0.9 km), which confirmed distance as the main factor that directly influences the mode of AC.

PA-levels also seem to be associated with the mode of commuting, according to our results. University Chilean students with higher PA-levels reported more active modes of commuting than those with lower PA-levels, who presented more passive modes of commuting to university. When we analyzed this association separating those living in shorter, medium, and further distances from university, such association only remained between medium and low PA-levels for those living in medium distances (i.e., from 2 km to 5 km). Consequently, the effect of walking to university could contribute to increasing the PA-levels when walking distances from 2 to 5 km, but not when walking shorter distances. This association may not occur for long distances since more than 5 km is not considered in the scientific literature as a walkable distance to be an active commuter in the youth population [41]. In addition, the low sample of students who are active and live further than 5 km in the current study (i.e., 10.2%; *n* = 28) may hamper finding significant results. There is a lack of studies addressing the threshold distance that university students are willing to walk to university. Furthermore, under our knowledge, there are no studies linking PA-levels with the mode of commuting to university in Chile. A recent study about PA patterns measured through IPAQ (extended version) in the Southern Cone of Latin America [42] revealed that the lowest prevalence of PA related to transportation was observed firstly in Barrios Blancos (Uruguay), and then in Temuco (Chile). According to our results, a European study conducted in adolescents [43] observed a positive association between higher PA-levels and AC measured by accelerometers (objective measure) and self-report measures. Similar results have been reported in young population from USA [44], in workers from Swiss alpine communities [45], and in the adult population from Brazil [46]. However, there is previous evidence among Spanish university students of the lack of association between ACU and the overall PA [19]. Further studies with objective measures are needed to delve into the relationship between the levels of PA and the mode of commuting to university in Chilean students. Additionally, it is important to consider that the transition to the adulthood is related to decrease the active mode of commuting and increase the use of public transport [8], which may reduce the total PA-levels. However, the bus, as a public transport, has been cited as a way to help individuals to incorporate regular PA into their day since people have to walk to the public transport stops [47,48]. A study in Spanish university students estimated that those using public transportation as a mode of commuting to university could incorporate a total of 96 min/week of extra PA, which could translate into 1.5 kg less in weight gained per year. On the other hand, drivers could only spend 16 min/week in AC, or lose 0.2 kg/academic year. Therefore, choosing one active mode for commuting to university, even public transport such the bus, can make a large difference in annual energy expenditure [18]. However, in our study, we did not investigate in depth the walking distance between the bus stop and university or home [22]. This could be an interesting point to support the medium and high PA-levels reported by the Chilean students.

Results of our study must be viewed in the light of methodological limitations. One limitation of this study is its cross-sectional nature; consequently, we may not confirm whether the modes of commuting determine the PA-levels or vice-versa. A random sampling of the universities was not performed, so the study sample is not likely to be representative. Measurements used were self-reported. The IPAQ could present problems for the responders to provide exactly the hours and minutes for sitting and walking [49], which could lead to a weakness for the estimating of low-physical-activity. However, the IPAQ short form “Past 7 days” is the preferred measure, owing to its excellent test-retest reliability over 7 days, compatibility across cultures within non-English speaking countries, and this, along with its short format, makes it feasible for research and clinical practice. Additionally, an adequate sample size of over 100 participants is recommended, a criterion that has remained considerably achieved in our study [50]. Finally, it should be taken into account that under our knowledge, this is the first study analyzing Chilean’s university students commuting behaviors and its relationship with socio-demographic factors and PA-levels.

## 5. Conclusions

University students from Valparaíso (central coast of Chile) mainly used public transportation (i.e., bus) as the main mode of commuting to university. Students living closer to university and more physically active were more likely to use active modes of commuting to university. Our results seem to indicate that to be an active commuter to and from university in distances between 2–5 km could contribute to having medium PA-levels. Efforts to increase ACU are expected to have a long-ranging health benefit, however, the mode of commuting is a multifactorial behavior, thus a wide range of factors are associated, such as distance, built environment, environmental awareness, the accompaniment of friends, or optimal access (proximity to bus stops). Therefore, further studies, including these factors and objective measures, would be the key to propose successful strategies increasing the overall PA-levels and promoting health in the university communities through the ACU.

## Figures and Tables

**Table 1:** Descriptive data of the participants.

Variables	All *N* (%) (*n* = 459)	Passive Commuters *N* (%) (*n* = 307)	Active Commuters *N* (%) (*n* = 152)	*p* Value Active Commuters vs. Passive Commuters
**Sociodemographic factors **				
Gender				
Male	147 (32.0)	104 (33.9)	43 (28.3)	0.227
Female	312 (68.0)	203 (66.1)	109 (71.7)	
Age (years) *	21.6 ± 2.4	21.9 ± 2.3	21.4 ± 2.8	0.694
*Age groups*				
18–20 years old	155 (33.9)	98 (31.9)	57(37.5)	0.404
21–23 years old	203 (44.3)	138 (45.0)	63 (42.8)	
>24 years old	100 (21.8)	71 (23.1)	29 (19.1)	
University Life (years) *	3.17 ± 1.7	3.17 ± 1.7	3.01 ± 1.8	0.142
University Life groups				
≤3 years at university	307 (66.9)	116 (37.8)	67 (44.1)	0.234
>3 years at university	152 (33.1)	187 (60.9)	85 (55.9)	
Distance to university (km) **	7.6 (2.8, 17.2)	10.7 (6.0, 18.3)	1.3 (0.8, 3.4)	<0.001
Distance to university groups				
> 5 km	272 (62.1)	244 (79.5)	28 (21.4)	<0.001
2–5 km	74 (20.4)	56 (18.2)	18 (13.7)	
≤2 km	92 (21.0)	7 (2.3)	85 (64.9)	
Socioeconomic Status				
High	216 (47.0)	150 (48.9)	66 (43.4)	0.547
Medium	195 (42.5)	126 (41.0)	69 (45.3)	
Low	48 (10.5)	31 (10.1)	17 (11.2)	
**Physical Activity Patterns**				
MET-min/week *	2231.4 ± 1898.0	2135.9 ± 1750.4	2355.4 ± 2198.4	0.082
Seated-min/week *	2962.2 ± 1787.1	2942.6 ± 1857.3	3009.7 ± 1715.1	0.532
Physical Activity Level				
Low	140 (30.5)	100 (32.6)	40 (26.3)	0.203
Medium	176 (38.3)	119 (38.8)	57 (37.5)	
High	143 (31.2)	88 (28.7)	55 (36.2)	

* Mean ± Standard Deviation. ** Kilometers of usual distance to and from university are expressed as median (25th percentile, 75th percentile). MET: metabolic equivalent of task.

**Table 2:** Associations (odds ratio, OR, and 95% confidence intervals, 95% CI) between active commuting to university and sociodemographic factors and physical activity.

Variables	Active Commuting to University				
	*n*	OR	95% CI		*p*
**Sociodemographic Factors**					
Age groups					
18–20 years old	155	*Reference*			
21–23 years old	203	1.173	0.641	2.145	0.604
>24 years old	100	0.984	0.484	2.002	0.965
University life groups					
≤3 years at university	183	*Reference*			
>3 years at university	272	1.073	0.882	0.938	0.799
Distance groups					
> 5 km	277	*Reference*			
2–5 km	86	4.424	2.443	8.011	<0.001
≤2 km	96	143.052	55.154	371.030	<0.001
Socioeconomic status					
Low	48	*Reference*			
Medium	195	0.939	0.454	1.940	0.865
High	216	0.742	0.361	1.523	0.416
**Physical activity patterns**					
Physical Activity Levels					
Low	140	*Reference*			
Medium	176	1.446	0.864	2.421	0.160
High	143	1.880	1.880	1.094	0.022

Analysis adjusted for distance from home to university (except for the distance groups analysis) and gender.

**Table 3:** Odds ratio (OR) for active commuting to the university by physical activity levels categorized by distance from home to university.

Physical Activity Levels	Active Commuting to University				
	*n*	OR	95% CI		*p*
≤2 km					
Low	33	*Reference*			
Medium	27	1.824	0.234	14.235	0.566
High	36	3.589	0.249	51.761	0.348
2–5 km					
Low	22	*Reference*			
Medium	41	5.244	1.358	20.246	0.016
High	23	1.153	0.248	5.350	0.856
>5 km					
Low	85	*Reference*			
Medium	108	1.555	0.549	4.404	0.406
High	81	1.876	0.654	5.380	0.242

Analysis adjusted for university and gender.
